# First-in-human e-Flash radiotherapy using a modified conventional C-arm linear accelerator

**DOI:** 10.1016/j.ctro.2025.101047

**Published:** 2025-09-23

**Authors:** Jens von der Grün, Riccardo Dal Bello, Serena Psoroulas, Jerome Krayenbuehl, Debra Fesslmeier, Egle Ramelyte, Joanna Mangana, Wendy Smith, Marta Vilalta, Lena Tirpak, Ricky A. Sharma, Matthias Guckenberger, Stephanie Tanadini-Lang, Panagiotis Balermpas

**Affiliations:** a Department of Radiation Oncology, University Hospital Zurich, University of Zurich, Zurich, Switzerland; b Department of Dermatology, University Hospital Zurich, University of Zurich, Zurich, Switzerland; c Varian Medical Systems, Inc., Palo Alto, California, United States

**Keywords:** Flash-RT, UHDR, Electrons, Malignant melanoma, Clinical translation, Dosimetry

## Abstract

**Background:**

The FLASH effect is considered being the widening of the therapeutic window at (ultra-)high dose rates due to sparing of normal tissues while preserving tumor response. Our goal was to provide first data on the safety in treating a patient using a conventional linear accelerator converted to deliver 9 MeV UHDR electron beams in the research setting.

**Material & methods:**

A conventional Varian TrueBeam linac was converted in the research setting to become capable of delivering 9 MeV UHDR electron beams. A phase I trial protocol was approved by the local authorities (NCT06549439). One patient with ≥ 1 melanoma (sub)-cutaneous lesion(s) was treated with 3x 9 Gy. The first two fractions were applied via e-Flash and the third via conventional electrons.

**Results:**

Here we present the experience of the first patient treated with e-Flash in a modified conventional linac. The e-Flash fractions were successfully delivered according to the protocol. The film in-vivo dosimetry confirmed correctness of the delivered dose. A good tumor response without severe or unexpected toxicity was observed up to six weeks after treatment.

**Conclusion:**

We demonstrate first-in-human application of e-Flash delivered by a modified conventional linear accelerator. Conversion of conventional linacs into e-Flash systems will accelerate testing Flash radiotherapy in clinical trials.

## Highlights

• First treatment of a patient by e-Flash radiotherapy with a modified conventional C-arm linear accelerator.
• Detailed technical description including hardware, treatment planning, quality assurance, and dosimetry summary.
• Overview of ongoing clinical trials utilizing Flash radiotherapy.

## Introduction

Radiotherapy is one of the pillars of cancer treatment [1] with normal tissue toxicity remaining the dose-limiting factor [2]. For radiation to be therapeutically effective, tumor control should occur at a dose lower than the level causing severe toxicity. This “therapeutic window” refers to the dose range between the probability of normal tissue complications and the probability of tumor control [3]. Expanding this window is a main goal of research in radiation therapy (RT).

In preclinical studies, an expansion of the therapeutic window was observed using ultra-high dose rates (UHDR). This requires roughly > 40 Gy/s, though the exact definition of the physics parameters remains open [[4], [5], [6]]. This biological effect has been termed 'Flash Effect’, confirmed by a large number of experiments [[7], [8], [9]].

The first clinical treatments using UHDR electrons have been delivered on specialized research devices [10], limiting broader clinical translation. In this first-in-human study, we investigate the feasibility of treating a patient using UHDR electrons on a conventional linear accelerator. Conventional linacs are widely available in radiation therapy centers worldwide, making the implementation of Flash-RT more accessible. With dedicated technical modifications [11], standard clinical accelerators can be adapted for UHDR electron delivery, as an attractive alternative to dedicated platforms.

Here we report the first-in-human experience of delivering Flash-RT using a modified conventional C-arm linear accelerator, opening the possibility for faster adoption of Flash-RT in the clinic.

## Materials and methods

### Trial protocol summary

**Fig. 1:**
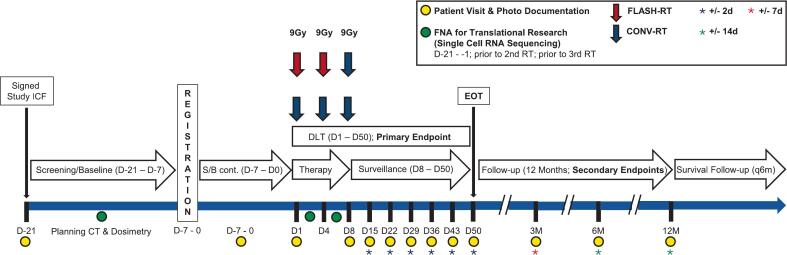
Study scheme. Abbreviations: RT − Radiotherapy, CONV − Conventional, D − Days, M − Months, FNA − Fine-needle aspiration, RNA − Ribonucleic acid, DLT − Dose-limiting toxicity, S/B − Screening/Baseline, EOT − End of treatment, ICF − Informed consent form.

The patient was treated within a phase I clinical trial at the University Hospital Zurich, Switzerland (Flash-Skin I, NCT06549439, Fig. 1), approved by the local ethics committee (BASEC-Nr.: 2024-D0073).

In the primary trial cohort, six patients with ≥ 1 melanoma (sub)-cutaneous lesion(s) will be treated with 3x 9 Gy (2x fractions/ week; α/β(3): EQD2: 64.8 Gy; α/β(10): EQD2: 42.75 Gy) [12,13]. Detailed inclusion and exclusion criteria are shown in Supplementary Table 1. A minimum of one lesion will be treated with two fractions of Flash-RT (2x 9 Gy) and one of Conv-RT (1x 9 Gy) as experimental treatment and one or more other lesions will be treated with conventional RT (Conv-RT, 3x 9 Gy) as “internal” control. Patients with a single lesion can be included in the trial. Following dosimetry of the two Flash-RT fractions, the third fraction will be applied by Conv-RT, adjusted to compensate for potentially lower or higher doses applied with Flash-RT before if necessary. This was introduced as a safety measure, as the feedback loops controlling the along-beam internal dosimetry of the UHDR linac are disabled. This approach ensures that every lesion is ultimately treated with precisely a total reference dose of 27 Gy (±5%). Although this approach could bias the clinical results regarding treatment effect it was deliberately chosen as an extra safety measure, as the modified platform is not approved for clinical use and caution was warranted. Moreover, an additional goal and secondary endpoint was to demonstrate technical feasibility.

Primary endpoint of the trial is safety. Patients will be monitored before each fraction and then weekly for 6 weeks, then every 3 months. Safety will be confirmed if a maximum of 2 out of 6 patients develop dose limiting toxicity (DLT) in the primary patient cohort. Skin toxicity greater than grade III at any time point or clinically relevant skin toxicity equal to grade III which lasts for > 2 weeks (CTCAE V5.0) during and within six weeks (day 42) after Flash-RT will be considered as DLT. Secondary endpoints include local response, symptom relief, late adverse effects, and feasibility.

### Patient and tumor characteristics

The female patient was diagnosed with a melanoma of the right foot at the age of 68 years. The primary tumor and inguinal lymph nodes were resected in September 2018 (pT3a, pN3c, M0; BRAF non-V600 mutation HLA 02:01 pos). Then 14 cycles of adjuvant Nivolumab followed. Upon progression, the patient received combined Nivolumab/Ipilumumab. The therapy was stopped due to progression. From 05/2022 to 01/2024, the patient underwent resection of three metastases at the right leg and right inguinal region. In June 2023, the patient received postoperative RT with 20x 2.4 Gy to the inguinal region and a boost of 4x 2.4 Gy for R1. A new line of treatment was started in July 2024 with Talimogen Laherparepvec (T-VEC) and stopped after further progression. The patient was then treated with Tebentafusp from 12/2024 until 02/2025 within a trial (NCT05549297) which was discontinued at patient's request. In March 2025, a fourth-line with Nivolumab/Relatlimab was started, ongoing at the time-point of irradiation. Following multidisciplinary board decision, the patient received palliative RT within the Flash-Skin I trial with 3x 9 Gy twice per week for an exophytic, bleeding melanoma lesion at the right lower leg in March 2025.

### Hardware

This study employed Flash for TrueBeam v2.7.5 SN1001 (Varian Medical Systems, Inc.), a converted linac now capable of delivering 9 MeV UHDR electron beams. The technical conversion included hardware (e.g. thinner scattering foils) and firmware (e.g. pulse counting) modifications, as previously reported [11]. The feedback loops controlling the along-beam internal dosimetry were disabled, i.e. the linac ion chamber does not control the total delivered dose. The firmware was designed such that the number of pulses to be delivered was specified by the number of monitor units (MU), i.e. 1 MU = 1 pulse. Redundant safety loops were introduced to avoid delivery of an excessive number of pulses, which were: independent pulse counting with termination capability by the Beam Generation and Monitoring subsystem (BGM), independent beam termination capability by the Stand subsystem (STA) based on the beam-on timer and maximum allowed pulses set to 20 for plans delivered in treatment mode.

The system was designed to deliver the 9 MeV UHDR beam at the isocenter with a dose per pulse of 1.08 Gy at Dmax in water with maximum allowed field size of 10x10 cm^2^. The pulse repetition frequency was 200 Hz and pulse length 4.5 µs, which led to average and instantaneous dose rates of 216 Gy/s and 2.4·10^5^ Gy/s, respectively. Further beam characteristics are reported in Supplementary Table 2 [14]. Functionalities such as cone-beam computed-tomography (CBCT) acquisition and matching, patient positioning with the six degrees of freedom (6 DoF) couch, beam delivery in treatment mode and use of conventional electron tubes were maintained.

### Treatment planning

The treatment planning was performed adopting a standard clinical workflow: a planning CT was acquired, the contouring of was performed with Aria v16.1 (Varian), the treatment plan manually optimized in Eclipse v16.1, the dose calculated with Electron Monte Carlo (EMC) v16.1 and the plan was transferred to the linac using the patient scheduling tools. This process was performed on a test server (T-Box), which was dedicated entirely to Flash-RT. The server was connected exclusively to the modified first TrueBeam (SN1001): “Flash for Truebeam v2.7.5”. The third fraction delivered with conventional electron beams was planned in the clinical Aria v16.1. It should be noted that while the EMC version was the same in the clinical Aria and in the T-Box, the latter was fine-tuned to the beam characteristics of the UHDR beam. In-depth and lateral dose profiles were fed and fitted with EMC.

**Fig. 2:**
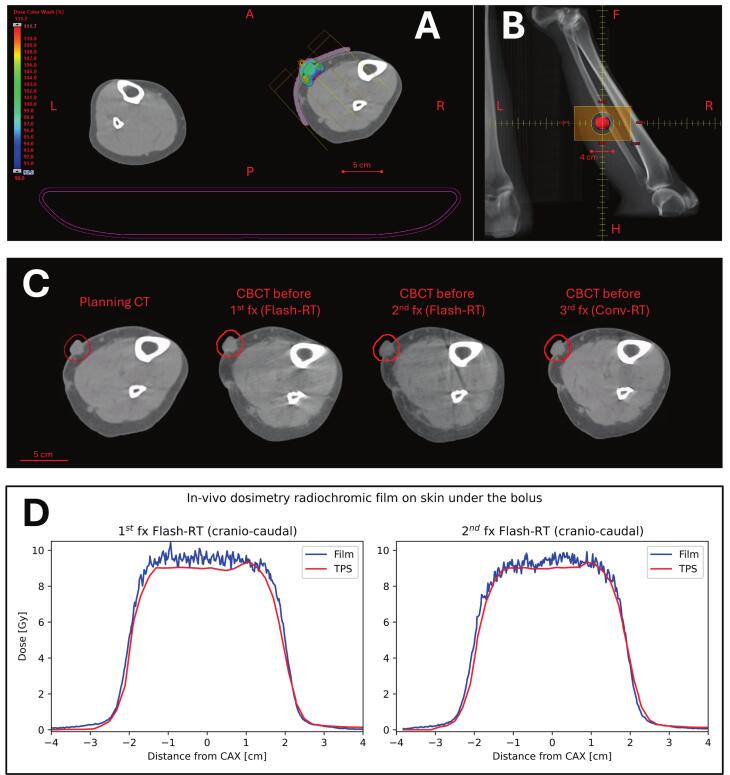
Patient positioning and dosimetry. A: Axial view of the treated planning target volume (PTV, red); B: Beam's eye view; C: Image-guidance for each fraction implementing cone-beam computed-tomography; D: Dosimetric results for each e-FLASH fraction confirming correct dose application for the same patient; Abbreviations: RT − Radiotherapy, CONV – Conventional, CT – Computed-tomography, FX – Fraction of Radiotherapy. (For interpretation of the references to colour in this figure legend, the reader is referred to the web version of this article.)

Both treatment plans were based on a round field of diameter 4 cm, which irradiated a 10 cm^3^ PTV (Fig. 2A-B). Maximum PTV depth from the skin was 2.0 cm and a 0.5 cm thick water-equivalent bolus was applied. The gantry was set to 315°, collimator 0° and no couch rotations were performed. The two Flash-RT fractions delivered 10 pulses each and the total delivery time was 45 ms per fraction. The Conv-RT delivered 1160 MU in 1 min 10 s. Both plans respected the protocol coverage requirements (Table 1).

**Table 1:** Dosimetric parameters of the Conv-RT and Flash-RT plan.

Dosimetric parameter	Conventional RT	FLASH RT	Required
PTV D90%	97.99 %	95.4 %	> 80 %

PTV D2%	110.97 %	107.66 %	< 115 %

GTV D95%	97.64 %	96.64 %	N.A.

### Quality assurance and dosimetry summary

Quality assurance was performed according to national guidelines (SSRMP Rec. 11 and 16), international recommendations [15], vendor-recommended actions, protocol-defined measurements and standard operational procedures of the department. A detailed overview was provided elsewhere [16]. We highlight here the beam-monitoring procedures: daily output and symmetry pre-and post-treatment; in-vivo dose measurements with radiochromic EBT3 film; along-beam output monitoring with passive detectors and along-beam pulse counting with active time-resolved detector. The detectors used have been previously characterized [17].

## Results

The two Flash-RT fractions were successfully delivered on TrueBeam v2.7.5. The third fraction (Conv-RT) was delivered at the TrueBeam SN3232. The CBCTs for patient positioning are reported in Fig. 2C.

The UHDR daily output was on average + 0.8 % to the nominal value (single detector maximum deviation within ± 4.3 %) for the first and + 0.5 % (max. deviation ± 3.9 %) for the second Flash-RT fraction, as measured with four independent detectors before and after delivery. The symmetry was within ± 2.3 % for the first and ± 4.2 % for the second Flash-RT fraction, as measured with four independent off-axis points before and after delivery. The film in-vivo dosimetry under the bolus confirmed correctness (Fig. 2D). The 2D gamma analysis (4 %/3mm, dose threshold = 10 %) returned 97.7 % for the first Flash-RT fraction and 97.6 % for the second Flash-RT fraction. The output during RT delivery of the UHDR linac measured with OSLD was + 0.11 % and −0.22 % compared to nominal for the first and second fraction, respectively, respecting study feasibility requirements for single Flash-RT fractions. The daily output of the conventional linac the day of the third Conv-RT fraction was + 0.54 % compared to nominal. The total dose delivered was therefore + 0.14 % compared to the planned dose, respecting the study feasibility requirements. Finally, the active detectors measured independently from the linac the number of pulses and total delivery time, confirming 10 pulses and 45 ms for both Flash-RT fractions.

**Fig. 3:**
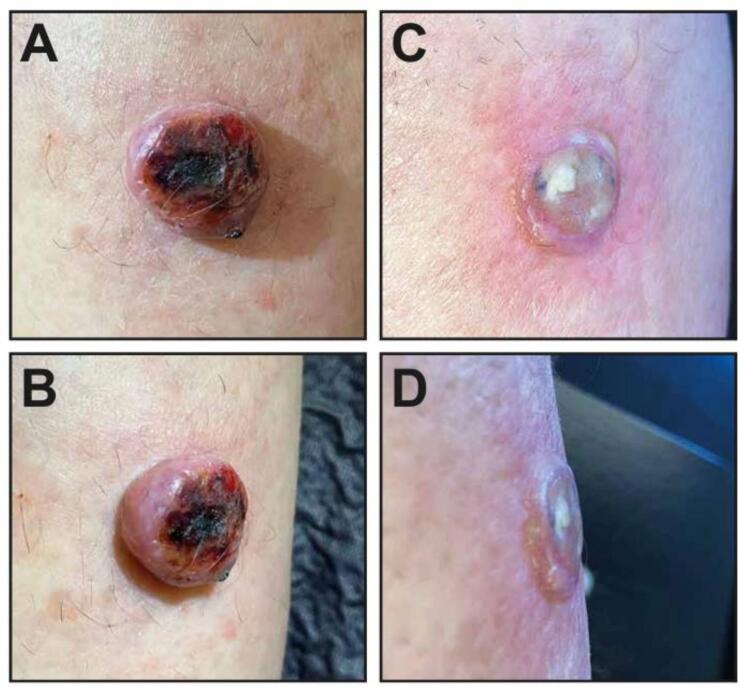
Exophytic melanoma lesion prior to FLASH-radiotherapy (A-B) and six weeks after completion of radiotherapy (C-D).

The patient tolerated the treatment well, no pain, no moist desquamation, or any other sequelae or discomfort were reported during treatment and up to 6 weeks after radiotherapy. The maximum reaction of the surrounding skin was grade I radiodermatitis. Already after one week the bleeding episodes became rare and after two weeks the tumor began to shrink. A good objective remission was observed at the end of the initial follow up phase (Fig. 3).

## Discussion

Flash-RT aims to spare normal tissues while preserving the therapeutic effect. Preclinical studies have shown evidence for the flash-effect in cell culture and animal studies [9]. To the best of our knowledge, this study reports on the first application of electron Flash-RT delivered by a clinical C-arm linac modified for UHDR.

To this day only very few, veterinary trials have been conducted on modified clinical linacs able to produce Flash-RT. Konradsson et al. [18] reported on the feasibility of irradiating 10 canine patients for various malignancies on a modified Elekta Precise linear accelerator (Elekta AB, Stockholm, Sweden) described [19]. Recently, another trial demonstrated again the safety of the approach in a cohort of 14 dogs, using the same platform and single-dose Flash [20]. All of the patients showed partial or complete response. Previously, only dedicated prototypes, able to produce electron beams up to thousands Gy/s, have been used for this purpose, e.g. in the seminal trial of Vozenin et al., describing the response of 6 cat patients treated for squamous cell carcinoma of the nasal planum [21].

We have shown that the Flash-RT could be planned and delivered with a workflow analogous to the clinical workflow for electron treatments. Due to the investigational nature of the device, the Flash-RT fractions required a larger QA effort, which confirmed the precision of the delivery (gamma analysis agreement > 97 %, output deviation < 5 %).

As Flash-RT requires machine and/or monitoring configurations outside the approved specifications for radiotherapy, treatments so far were performed on experimental or dedicated, investigational devices. In 2018, a patient with CD30 + T-cell cutaneous lymphoma was the first treated with Flash-RT. Treatment was performed using an Oriatron eRT6 5.6-MeV Linac at Lausanne University Hospital, a prototype provided by PMB/Alcen (Peynier, France) [22]). A skin lesion was treated with a single fraction of 15 Gy in 90 ms. Acute adverse effects were mild and the treatment resulted in complete response [10]. Later, the same patient was treated again with Flash-RT [23]. Since then, five prospective clinical trials have been launched (Table 2). FAST-01 (phase I, Cincinnati Children’s/UC Health Proton Therapy Center) was the only one which has reported results; trial endpoints were feasibility, toxicity, and pain relief. Adverse events were mild and consistent with Conv-RT [24]. Ongoing trials are mainly phase I, with only one phase II trial, and all (including our own) have toxicity as endpoint. Since they all use investigational devices (one proton gantry, one IORT electron linac, and the C-arm gantry linac used here), feasibility and safety is carefully monitored and reported. Results published so far are satisfactory in terms of precision, accuracy and stability, especially considering that pulse monitoring in the investigated linacs is still limited and under development [25].

**Table 2:** Overview of ongoing, prospective clinical Flash-RT trials.

**Institute /** **Trial Name /** **NCT Identifier**	**Investigational device**	**Phase/** **endpoint**	**Clinical setting**	**Fractionation**	**N**
**Single-arm studies:**					

FAST-01 (Cincinnati Children’s/UC Health Proton Therapy Center, USA)	Varian ProBeam (proton gantry)	Phase I Toxicity and Feasibility − completed	Painful bone metastases of the extremities	1x8 Gy	10

FAST-02 (Cincinnati Children’s/UC Health Proton Therapy Center, USA)	Varian ProBeam (proton gantry)	Phase I Toxicity and Efficacy	Painful bone metastases of the ribs, clavicles, scapulae, or sternum	1x8 Gy	10

IMPULSE (University Hospital of Lausanne (CHUV), Switzerland)	Mobetron (IORT electrons)	Phase I Maximum Tolerated Dose	melanoma skin metastases	Single fraction, dose escalation from 22 to 34 Gy in 2 Gy interval	46

Flash-Skin 1 (University Hospital Zurich (USZ), Switzerland)	Varian Truebeam (C-arm gantry linac, electrons)	Phase I Toxicity (and Efficacy)	malignant melanoma	2x9 Gy FLASH-RT + 1x9 Gy CONV-RT	6–10

**Randomized studies (FLASH-RT vs. CONV-RT):**					

LANCE (University Hospital of Lausanne (CHUV), Switzerland)	Mobetron (IORT electrons)	Phase II Selection Trial Toxicity and Feasibility	localized cutaneous squamous cell carcinoma (cSCC) or basal cell carcinoma (BCC)	1x22 Gy (T1 lesion ≤ 2 cm in diameter) or 5x6 Gy (T2: 2 cm < lesion ≤ 4 cm)	60

In our study, we show how a conversion of a clinical, C-arm gantry linac can be safely performed. Given the wide availability of C-arm gantry linacs this might pave the way for a wider adoption of Flash-RT.

## Conclusion

Electron Flash-RT was successfully applied for the first time in a clinical setting by a conventional C-arm linac following dedicated technical modifications. The results of the ongoing study will confirm feasibility of the technique, which could facilitate a more rapid adoption of the UDHR-technology in clinical practice.

## CRediT authorship contribution statement

**Jens von der Grün:** Conceptualization, Formal analysis, Funding acquisition, Investigation, Methodology, Writing – original draft, Writing – review & editing. **Riccardo Dal Bello:** Data curation, Formal analysis, Funding acquisition, Investigation, Methodology, Software, Validation, Visualization, Writing – original draft, Writing – review & editing. **Serena Psoroulas:** Data curation, Investigation, Methodology, Validation, Visualization, Writing – original draft, Writing – review & editing. **Jerome Krayenbuehl:** Data curation, Investigation, Validation, Visualization, Writing – original draft, Writing – review & editing. **Debra Fesslmeier:** Data curation, Investigation, Validation, Writing – original draft, Writing – review & editing. **Egle Ramelyte:** Data curation, Investigation, Writing – review & editing. **Joanna Mangana:** Data curation, Investigation, Supervision, Writing – review & editing. **Wendy Smith:** Methodology, Project administration, Resources, Software, Writing – review & editing. **Marta Vilalta:** Methodology, Project administration, Resources, Software, Writing – review & editing. **Lena Tirpak:** Methodology, Project administration, Resources, Software, Writing – review & editing. **Ricky A. Sharma:** Methodology, Project administration, Resources, Software, Supervision, Writing – review & editing. **Matthias Guckenberger:** Conceptualization, Funding acquisition, Project administration, Resources, Supervision, Writing – review & editing. **Stephanie Tanadini-Lang:** Conceptualization, Formal analysis, Funding acquisition, Supervision, Writing – original draft, Writing – review & editing. **Panagiotis Balermpas:** Conceptualization, Formal analysis, Funding acquisition, Investigation, Writing – original draft, Writing – review & editing.

## Ethics approval

The study was approved by the cantonal ethics committee of Zurich (BASEC-Nr.: 2024-D0073).

## Funding

The Flash-Skin I trial (NCT06549439) was funded by a research grant from Varian Medical Systems, Inc.

## Declaration of competing interest

The authors declare the following financial interests/personal relationships which may be considered as potential competing interests: JVDG and the department of Radiation Oncology received research grants from Varian/ Siemens Healthineers. RDB received research funding from the Inovationspool of the University Hospital Zurich. JM has intermittent project focused consultant/advisory relationships or has received speaker fees from Merck Sharp & Dohme, Novartis, Janseen, Bristol Myers Squibb and Pierre Fabre and has received travel support from Sunpharma, L’ oreal, Merck Sharp & Dohme, Bristol Myers and Squibb und Pierre Fabre outside of the submitted work. ER received research funding by Amgen; consulting or advisory roles for Sanofi/Regeneron and Amgen; honoraria from Pierre Fabre, Lilly, Galderma, MSD, Sanofi, and BMS GmbH & Co KG; Travel expenses from Amgen and Sanofi. WS, MV, LT, and RAS reported employment at Varian Medical Systems, Inc., the commercial sponsor of the study.

## References

[1] Mee T. (2023). The use of radiotherapy, surgery and chemotherapy in the curative treatment of cancer: results from the FORTY (Favourable Outcomes from RadioTherapY) project. Br J Radiol.

[2] Thariat J. (2013). Past, present, and future of radiotherapy for the benefit of patients. Nat Rev Clin Oncol.

[3] Baumann M., Petersen C. (2005). TCP and NTCP: a basic introduction. Rays.

[4] Montay-Gruel P. (2021). Hypofractionated FLASH-RT as an effective treatment against glioblastoma that reduces neurocognitive side effects in mice. Clin Cancer Res.

[5] Favaudon V. (2014). Ultrahigh dose-rate FLASH irradiation increases the differential response between normal and tumor tissue in mice. Sci Transl Med.

[6] Sørensen B.S. (2022). Pencil beam scanning proton FLASH maintains tumor control while normal tissue damage is reduced in a mouse model. Radiother Oncol.

[7] McGarrigle J.M., Long K.R., Prezado Y. (2024). The FLASH effect—an evaluation of preclinical studies of ultra-high dose rate radiotherapy. Front Oncol.

[8] Toschini M. (2025). Medical physics dataset article: a database of FLASH murine in vivo studies. Med Phys.

[9] Vozenin M.-C., Bourhis J., Durante M. (2022). Towards clinical translation of FLASH radiotherapy. Nat Rev Clin Oncol.

[10] Bourhis J. (2019). Treatment of a first patient with FLASH-radiotherapy. Radiother Oncol.

[11] Dal Bello R. (2023). Enabling ultra-high dose rate electron beams at a clinical linear accelerator for isocentric treatments. Radiother Oncol.

[12] Overgaard J., von der Maase H., Overgaard M. (1985). A randomized study comparing two high-dose per fraction radiation schedules in recurrent or metastatic malignant melanoma. Int J Radiat Oncol Biol Phys.

[13] Barker C.A., Lee N.Y. (2012). Radiation therapy for cutaneous melanoma. Dermatol Clin.

[14] Tobias Böhlen T. (2024). Recording and reporting of ultra-high dose rate “FLASH” delivery for preclinical and clinical settings. Radiother Oncol.

[15] Garibaldi C. (2024). Minimum and optimal requirements for a safe clinical implementation of ultra-high dose rate radiotherapy: a focus on patient's safety and radiation protection. Radiother Oncol.

[16] dal Bello R. Technical preparation for a phase i clinical study of e-Flash radiotherapy for palliative treatment of superficial skin lesions of malignant melanomas. 2024 [cited 2025 May 23rd]; Available from: https://scholar.google.com/citations?view_op=view_citation&hl=en&user=Dl5Wt6QAAAAJ&sortby=pubdate&citation_for_view=Dl5Wt6QAAAAJ:GnPB-g6toBAC.

[17] Bossin L. (2024). Performance of a BeO-based dosimetry system for proton and electron beam dose measurements. Radiat Meas.

[18] Konradsson E. (2021). Establishment and initial experience of clinical FLASH radiotherapy in canine cancer patients. Front Oncol.

[19] Lempart M. (2019). Modifying a clinical linear accelerator for delivery of ultra-high dose rate irradiation. Radiother Oncol.

[20] Gjaldbæk B.W. (2024). Long-term toxicity and efficacy of FLASH radiotherapy in dogs with superficial malignant tumors. Front Oncol.

[21] Vozenin M.C. (2019). The advantage of FLASH radiotherapy confirmed in mini-pig and cat-cancer patients. Clin Cancer Res.

[22] Jaccard M. (2018). High dose-per-pulse electron beam dosimetry: commissioning of the Oriatron eRT6 prototype linear accelerator for preclinical use. Med Phys.

[23] Gaide O. (2022). Comparison of ultra-high versus conventional dose rate radiotherapy in a patient with cutaneous lymphoma. Radiother Oncol.

[24] Mascia A.E. (2023). Proton FLASH radiotherapy for the treatment of symptomatic bone metastases: the FAST-01 nonrandomized trial. JAMA Oncol.

[25] Gonçalves Jorge P. (2025). Machine stability and dosimetry for ultra-high dose rate FLASH radiotherapy human clinical protocol. J Appl Clin Med Phys.

