# Supplementary material for: First-in-human e-Flash radiotherapy using a modified conventional C-arm linear accelerator
Source: Clin Transl Radiat Oncol. 2025 Sep 23;56:101047. doi: 10.1016/j.ctro.2025.101047 (PMC12509727; doi:10.1016/j.ctro.2025.101047)
Supplement: Supplementary Data 2 [file mmc2.docx]

**Supplementary Table 1:** Trial Inclusion and Exclusion Criteria.

| **Eligibility Criteria** | |
| --- | --- |
| **Inclusion Criteria** | 1.     Signed study Informed Consent Form.  2.     Males and females, age ≥ 18 years, no upper age limit.  3. Patients with metastatic melanoma and ≥ 1 skin/subcutaneous metastases (clearly definable in clinical examination: largest dimension of ≥ 5mm and ≤ 55 mm; ≤ 2.8 cm thickness (caliper-based measurement); volume ≤ 100ccm) with an indication for palliative radiotherapy of ≥ 1 skin/subcutaneous metastases according to the multidisciplinary tumorboard.  4.  The treated lesions must be at least 5 cm apart from each other, if applicable.  5.      Lesions located on the scalp can be treated.  6.      ECOG 0-2.  Note: Patients may receive concurrent standard of care systemic treatment. |
| **Exclusion Criteria** | 1.     Previous radiotherapy of the target lesions.  2.    Ulcerated lesions may not be treated within the study. Patients may have ulcerated tumor lesions besides those selected for treatment within the trial.  3.   Lesions, for which a homogeneous dose distribution inside the tumor D95%> 95% - D2% <107% for the PTV (acceptable deviation D90%> 80% – D2% <115%) in the treatment planning system cannot be achieved.  4.   Lesions should not be located on the face. Lesions on the forehead located cranially from a line situated 1 cm above the eyebrows can be treated (=cranial of sinus frontalis).  5.      Lesions should not be located directly on genitals.  6.  Lesions with close proximity to air-filled cavities or air-filled, luminal organs (e.g. bowel). Close proximity is defined by intersection of the respective part of the organ at risk with the 80% isodose line of the lesion planned for radiotherapy.  7.      Women who are pregnant or breast feeding.  8.  Lack of safe contraception during the study, defined as: Female participants of childbearing potential and male participants with partner of childbearing potential, not using and not willing to continue using a medically reliable method of contraception during the study and up to 6 months after therapy, such as oral, injectable, or implantable contraceptives, or intrauterine contraceptive devices, or who are not using any other method considered sufficiently reliable by the investigator in individual cases.  9.     Known or suspected non-compliance, drug or alcohol abuse, inability to follow the procedures of the study, e.g. due to language problems, psychological disorders, dementia, etc. of the participant, previous enrollment into the current study.  10.    Enrollment of the investigator, his/her family members, employees and other dependent persons.  11.    Active infection including tuberculosis (clinical evaluation that includes clinical history, physical examination and radiographic findings, and TB testing in line with local practice), hepatitis B (known positive HBV surface antigen (HBsAg) result), hepatitis C. Patients with a past or resolved HBV infection (defined as the presence of hepatitis B core antibody [anti-HBc] and absence of HBsAg) are eligible. Patients positive for hepatitis C (HCV) antibody are eligible  only if polymerase chain reaction is negative for HCV RNA. Not adequately controlled HIV disease (HIV-viral load detectable).  12.  Other severe comorbidities or psychiatric disorders (e.g. myocardial infraction within 6 months prior to registration, permanent cardiac arrhythmia, COPD Gold IV, schizophrenia, ongoing alcohol abuse) that would, according to the evaluation of the investigator, limit compliance with study requirement, substantially increase the risk of incurring adverse events or compromise the ability of the patient to give written informed consent.  13.   History of sun hypersensitivity or photosensitive dermatoses including porphyria, systemic lupus erythematosus, Sjögren's syndrome, xeroderma pigmentosum, polymorphous light eruptions.  14.      Concomitant auto-immune disease with skin lesions.  15.      Concomitant use of radio-sensitizer drug.  16.   Current, recent (within 10 days prior to start of study treatment), or planned participation in an experimental drug study (before end of treatment (EOT) visit).  17.   Concomitant use of systemic oncological treatment for another cancer than the metastatic melanoma. |

**Supplementary Table 1:** Trial Inclusion and Exclusion Criteria.
